# Genetic diversity and molecular analysis of metallo beta lactamases among imipenem resistant clinical isolates of *Pseudomonas aeruginosa* from Peshawar, Pakistan

**DOI:** 10.12669/pjms.37.7.4303

**Published:** 2021

**Authors:** Amjad Ali, Kafeel Ahmad, Shaista Rahat, Israr Ahmad

**Affiliations:** 1 Amjad Ali, PhD. Center of Biotechnology and Microbiology, University of Peshawar, Peshawar, Pakistan; 2 Kafeel Ahmad, PhD. Center of Biotechnology and Microbiology, University of Peshawar, Peshawar, Pakistan; 3 Shaista Rahat, PhD Scholar. Center of Biotechnology and Microbiology, University of Peshawar, Peshawar, Pakistan; 4 Israr Ahmad, PhD. Pakistan Health Research Council, Khyber Medical College, Peshawar, Pakistan

**Keywords:** *Pseudomonas aeruginosa*, Genetic diversity, RAPD, MBL, *blaIMP*, *blaVIM*, *blaNDM-1*

## Abstract

**Objectives:**

*Pseudomonas aeruginosa* is an opportunistic pathogen with remarkable adaptation ability to thrive in diverse environmental conditions. This study aimed at phenotypic and molecular analysis of metallo beta lactamases (*blaIMP, blaVIM, blaNDM-1 and blaSPM-1*) and genetic diversity analysis among imipenem resistant clinical isolates of *Pseudomonas aeruginosa*.

**Methods:**

This study was conducted from May 2017 to June 2018. The study included 187 *Pseudomonas aeruginosa* isolates collected from different clinical specimens from Peshawar, Pakistan. The isolates were analyzed for resistance to imipenem. Combined disc test (CDT) was then performed for phenotypic detection of metallo beta lactamases among imipenem resistant isolates of *Pseudomonas aeruginosa*. Molecular detection of metallo beta lactamases genes i.e. *blaIMP, blaVIM, blaNDM-1 and blaSPM-1* was analyzed through polymerase chain reaction. Genetic diversity was determined through RAPD-PCR.

**Results:**

MBL production was observed in 76% (n=19) isolates. The occurrence of MBL genes *blaIMP, blaNDM-1 and blaVIM* was 68% (n=17), 48% (n=12), and 4% (n=1) respectively. The *blaSPM-1* gene was not detected. High genetic diversity was observed in current study. Out of 182 isolates 171 isolates showed different RAPD profiles (93.95% polymorphism); 160 were unique RAPD strains and based on similarity coefficient ≥ 80%, 22 isolates were clustered into 11 distinct clones.

**Conclusion:**

A high prevalence of *blaIMP* and *blaNDM-1* among imipenem resistant isolates of *Pseudomonas aeruginosa* is alarming that calls for proper control and prevention strategies. RAPD technique was found to be a good genotyping technique when limited resources are available.

## INTRODUCTION

Extensive drug resistant strains of *Pseudomonas aeruginosa* have been reported from hospitals around the world.^1^ Resistance of *Pseudomonas aeruginosa* to different classes of antibiotics such as penicillin, cephalosporin, quinolone, aminoglycoside and carbapenem has been demonstrated.^2^ Metallo beta lactamases (MBL) are enzymes that catalyze the hydrolysis of broad-spectrum beta lactam antibiotics including carbapenems.^3^ Metallo beta lactamases (MBL) production has been reported from Brazil, Iran and India among carbapenem resistant clinical isolates of *Pseudomonas aeruginosa*.^4^-^6^ Among beta lactam antibiotics, carbapenems are the most effective against Gram negative and Gram-positive bacteria demonstrating broad range antibacterial activity.^7^ Resistant to carbapenems especially in Gram negative pathogens is global public health issue because of the spread of transferable carbapenemase encoding genes.^7^ Highest resistance was reported by Farooq et al., against imipenem in multi drug resistant clinical isolates of *Pseudomonas aeruginosa* from tertiary care hospital Karachi, Pakistan.^8^ Imipenem resistant pathogenic bacterial strains have been reported from Lahore and Quetta Pakistan.^9^,^10^ Imipenemase (IMP), Verona integron encoded metallo beta lactamase (VIM), New Delhi metallo beta lactamase (NDM), Sao Paulo metallo beta lactamase (SPM), Florence imipenemase (FIM) and Germany imipenemase (GIM) are various types of metallo beta lactamases that have been reported in carbapenem resistant isolates of *Pseudomonas aeruginosa*.^11^ Genes for MBLs are located on integrons, plasmids, transposons or on chromosomes.^11^ These specific genetic elements carry carbapenem and other antibiotics resistant determinants and thus confer multi drug resistance to *Pseudomonas aeruginosa*. These antibiotic resistant elements could be transferred to other Gram-negative bacterial strains and contribute towards spread of antimicrobial resistance rate making the treatment of infected patients complex.^11^ It is therefore, important to know about the epidemiology and resistance mechanisms of *Pseudomonas aeruginosa* in order to control and prevent multi drug resistant pathogenic strains to overcome possible health risks.^11^

Microbial typing is vital in order to determine the relationship between microbes.^12^ Knowledge about clonal relatedness between microbial strains is important to determine source, route of infection, detect cross transmission and confirm epidemic of pathogen.^12^ Microbial typing is also used in the recognition of virulent strains and to assess the effectiveness of control measures. Randomly amplified polymorphic DNA (RAPD) RAPD is a polymerase chains reaction (PCR) based genotyping method that requires short single arbitrary primers that could randomly amplify DNA and generate numerous discrete segments of DNA.^13^ RAPD demonstrates quick and efficient DNA polymorphism at large number of loci in genome of an organism. RAPD is simple, efficient, low-cost technique that needs no prior knowledge of target DNA sequence.^13^

No information is available on the occurrence of metallo beta lactamases and genetic diversity of *Pseudomonas aeruginosa* from Pakistan. Hence, this study aimed at analysis of MBL genes and genetic diversity of *Pseudomonas aeruginosa* isolates from the region.

## METHODS

### Bacterial isolates and identification

A total of 187 clinical isolates of *Pseudomonas aeruginosa* were collected from tertiary care hospitals (Khyber Teaching Hospital (KTH) and Hayatabad Medical Complex (HMC)) of Peshawar, Pakistan in 2017-2018. These included 74 isolates from pus, 34 isolates from urine, 24 isolates from sputum, 21 isolates from wound, 12 isolates from bronchial wash, 8 isolates from cerebrospinal fluid, six isolates from blood, 5 isolates from high vaginal swab and 3 isolates from diabetic foot. Patient’s informed consents and permission were obtained from the participating hospitals and study was ethically approved by Post Graduate Medical Institute (PGMI) Peshawar, KP Pakistan (Ref: 1046/PGMI, Dated: 03-05-2017). Cultures were inoculated on MacConkey agar (Oxoid, UK) and were incubated for 24 hours at 37 °C. Using standard procedures pure isolates were identified as *Pseudomonas aeruginosa* through morphological (Gram staining) and biochemical tests (catalase, oxidase, citrate utilization test, triple sugar iron test, indole test and nitrate reduction test).^14^

### Phenotypic detection of Metallo Beta Lactamases (MBL)

Twenty-five imipenem resistant isolates among the 187 isolates were subjected to phenotypic and genotypic detection of metalo lactamases. Combine disc test (CDT) was performed for phenotypic detection of MBL genes. Two imipenem discs (10 µg; Oxoid, UK) were placed 25 mm to 30 mm apart on surface of Muller Hinton agar (Oxoid, UK) inoculated with *Pseudomonas aeruginosa*. One imipenem disc was impregnated with 5 µl EDTA (0.5M, pH 8.0). Culture plates were incubated for 18 to 24 hours at 37 °C. Zones of inhibition were compared. The bacterial strain was considered as MBL positive if zone of inhibition of imipenem-EDTA disc was ≥ 7 mm than the zone of imipenem disc alone.^5^

### DNA extraction

GeneJET Genomic DNA purification kit (Thermo Scientific, Lithuania Cat. No. K0721) was used for DNA extraction. Recommended protocol was followed, and the quality of DNA was confirmed through gel electrophoresis using 0.8% agarose gel. The extracted DNA was preserved at −20°C for further use.

### Molecular detection of MBLs

Four metalo beta lactamases genes i.e., *blaIMP, blaVIM, blaNDM-1* and *blaSPM-1* were analyzed among 25 imipenem resistant isolates of *Pseudomonas aeruginosa* using previously reported primers and amplification profiles.^15^-^17^ The amplified PCR products were analyzed through gel electrophoresis and 100 base pair DNA ladder (Bioron, Cat. No. 304105) was used as size marker.

### RAPD-PCR

Genetic diversity was determined through RAPD-PCR using previously reported primer (RAPD 272) and procedures.^18^ Amplified products were analyzed through gel electrophoresis. The sizes of amplified DNA fragments were compared with 100 base pair (bp) DNA ladder (Bioron, Cat. No. 304105). RAPD PCR was repeated multiple times to ensure the reproducibility of the results and overcome the limitations of RAPD technique in a low-income setting.

### Statistical Analysis

Amplified bands through RAPD-PCR were scored as absent or present to generate bivariate (0-1) data for statistical analysis. Genetic diversity among the clinical isolates of *Pseudomonas aeruginosa* was determined using this bivariate data. Genetic distances among isolates were calculated using Nei and Li method by the formula D_xy_ = 1– (N_xy_) / (N_x_ + N_y_ – N_xy_) as reported.^19^ The term D_xy_ represents dissimilarity distance between two genotypes X and Y. Term N_xy_ represent the number of common bands present between two genotypes. The term N_x_ represent total number of bands present in genotype X and N_y_ shows total number of bands present in genotype Y. Using MEGA 7 (Molecular Evolutionary Genetics Analysis, version 7) software based on UPGMA (Unweighted Pair Group Method with Arithmetic mean) method dendrogram was generated from bivariate data obtained from RAPD analysis. Dendrogram was further analyzed for clonal relatedness among the clinical isolates of *Pseudomonas aeruginosa*. Distinct clones among the clinical isolates were defined based on similarity coefficient ≥ 80%.^20^

## RESULTS

**Fig.1:**
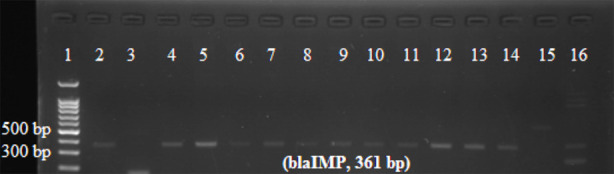
PCR amplification of blaIMP gene: Lane 1 (100 bp DNA ladder); Lanes 2, 4, 5, 6, 7, 8, 9, 10, 11, 12, 13, 14 and 16 are blaIMP positive samples with a 361 bp gene specific amplified fragment.

**Fig.2:**
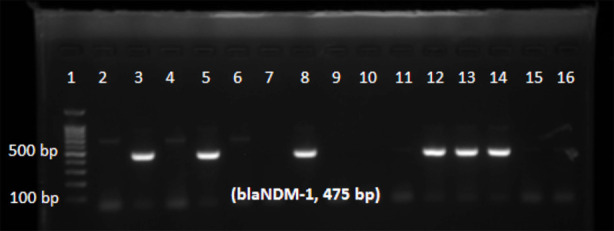
PCR amplification of blaNDM-1 gene: Lane 1 (100 bp DNA ladder); Lanes 3, 5, 8, 12, 13 and 14 contain blaNDM-1 (475 bp amplified fragment) positive samples of Pseudomonas aeruginosa.

**Fig.3:**
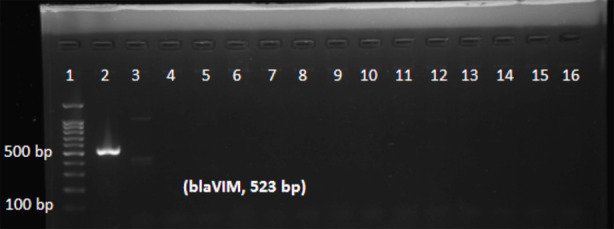
PCR amplification of blaVIM gene: Lane 1 (100 bp DNA ladder); Lane 2 contain blaVIM (523 bp amplified fragment) positive sample of Pseudomonas aeruginosa.

Of 187 isolates of *Pseudomonas aeruginosa*, 25 (13.36%) isolates were resistant to imipenem. Among these 25 imipenem resistant isolates, 19 (76%) isolates were phenotypically MBL producers (Table-I). On the basis of phenotypic tests, frequency of MBL producing isolates of *Pseudomonas aeruginosa* among different clinical specimens was: blood 5.26% (n=1), sputum 10.52% (n=2), wound 10.52% (n=2), bronchial wash 21.05% (n=4), urine 26.31% (n=5) and pus 26.31% (n=5) as given in Table-I. Metallo beta lactamases genes *blaIMP, blaNDM-1* and *blaVIM* were detected in 68% (n=17), 48% (n=12) and 4% (n=1) isolates respectively (Fig.1-3, Table-I). MBL gene *blaSPM-1* was not detected. The occurrence of Metallo beta lactamases genes correlated well with phenotypic detection of MBLs (Table-I).

**Table I:** Comparison of phenotypic MBL detection and occurrence of MBL genes (blaIMP, blaNDM-1, blaVIM and blaSPM-1) among imipenem resistant clinical isolates of Pseudomonas aeruginosa (+ means presence, − means absence).

*S. No*	*Isolate*	*Specimen*	*Phenotypic MBL status*	*Occurrence of MBL genes*			

				*blaIMP*	*blaNDM-1*	*blaVIM*	*blaSPM-1*
1	R9	Pus	−	−	−	−	−
2	R20	Blood	+	+	−	−	−
3	R31	Wound	+	−	+	−	−
4	R55	Wound	−	−	−	−	−
5	R59	Pus	+	+	+	−	−
6	R60	Bronchial Wash	+	+	+	−	−
7	R61	Wound	+	+	−	+	−
8	R62	Pus	+	+	+	−	−
9	R63	Urine	+	+	−	−	−
10	R64	Sputum	+	+	−	−	−
11	N27	Bronchial wash	+	+	+	−	−
12	N29	Bronchial wash	+	+	+	−	−
13	N35	Sputum	+	+	+	−	−
14	N39	Bronchial wash	+	+	+	−	−
15	N40	Urine	+	+	−	−	−
16	N55	Urine	+	+	+	−	−
17	N69	Sputum	−	−	−	−	−
18	N91	Wound	−	−	−	−	−
19	N95	Wound	−	−	−	−	−
20	N100	Urine	+	+	+	−	−
21	N102	Sputum	−	−	−	−	−
22	H38	Pus	+	+	−	−	−
23	H65	Urine	+	−	+	−	−
24	M26	Pus	+	+	−	−	−
25	K31	Pus	+	+	+	−	−

**Fig.4:**
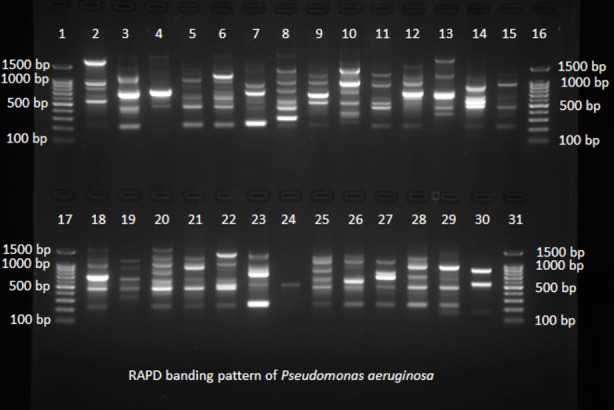
RAPD banding profile of *Pseudomonas aeruginosa* with RAPD primer 272: Lanes 1, 16, 17 and 31 = 100 bp DNA ladder; Lanes 2, 3, 4, 5, 6, 7, 8, 9, 10, 11, 12, 13, 14, 15, 18, 19, 20, 21, 22, 23, 24, 25, 26, 27, 28, 29 and 30 = RAPD banding pattern of *Pseudomonas aeruginosa* isolates.

**Fig.5:**
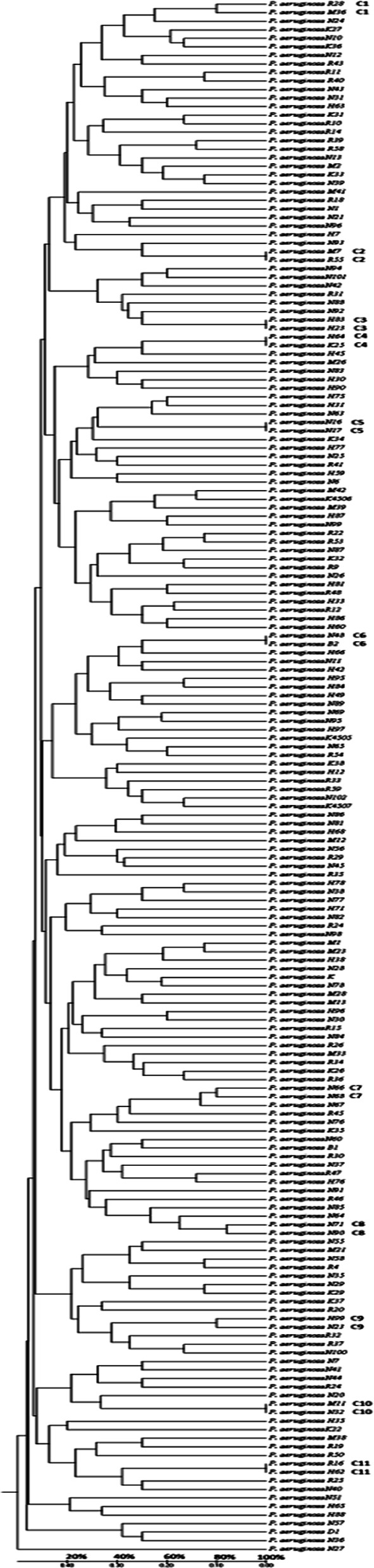
**Dendrogram:** Dendrogram generated using UPGMA method. Based on similarity coefficient of ≥80%, 11 clones were distinguished as C1= (R28, M36), C2= (M7, R55), C3= (H83, H23), C4= (H64, K25), C5= (N16, N17), C6= (N48, B2), C7= (N66, N68), C8= (N71, N90), C9= (H99, N23), C10= (M11, N32) and C11= (R16, H62).

Electrophoresis pattern of RAPD PCR products revealed a total of 31 distinct bands ranging in size from 150-1600 bp (Fig.4). Genetic dissimilarity distance values ranged from 0-1. Dendrogram of RAPD data is shown in Fig.5. Of the 182 isolates of *Pseudomonas aeruginosa* 171 isolates showed different RAPD profiles; 160 were unique RAPD strains and 22 isolates were clustered into 11 distinct clones. Genetic polymorphism was found to be 93.9%. Distinct clones among the isolates were defined based on similarity coefficient ≥ 80%. The 11 distinct clones were: R28, M36; M7, R55; H83, H23; H64, K25; N16, N17; N48, B2; N66, N68; N71, N90; H99, N23; M11, N32; R16, H62.

## DISCUSSION

In the current study, a high prevalence (76%) of MBLs was observed among imipenem resistant clinical isolates of *Pseudomonas aeruginosa*. Reports on MBL production in carbapenem resistant *Pseudomonas aeruginosa* have been reported from India (16.87%), Brazil (16.1%), Iran (60.4%) and Korea (41%).^4^,^21^-^23^ Patients infected with metallo beta lactamases producing bacteria are at escalated risk of treatment failure.^24^ Current study reported high prevalence of *blaIMP* (68%) as compared to previous findings from Iraq (50%), India (9%) and Egypt (4%) in carbapenem resistant isolates of *Pseudomonas* aeruginosa.^25^-^27^ High prevalence of *bla-NDM-1* (48%) was observed in current study in contrast to a previous report from India (10%).^26^ Low prevalence (4%) of *blaVIM* in this study is in contrast to previous findings by Mohanam et al. from India with 32% *blaVIM* prevalence and Hashem et al. from Egypt with 20% *blaVIM* prevalence.^26^,^27^ *BlaSPM-1* was not detected in current study. The gene has been reported previously from Iraq (16.6%) and Egypt (24%) in carbapenem resistant isolates of *Pseudomonas aeruginosa*.^25^,^27^ Rapid dissemination and increase in number of MBL producing *Pseudomonas aeruginosa* is worrisome and worldwide health problem.

Current study showed high genetic diversity (93.9% polymorphism) between *Pseudomonas aeruginosa* isolates. A study by Silva et al. also revealed high genetic diversity (89.6%) among 96 clinical isolates of *Pseudomonas aeruginosa* from Brazilian hospitals.^18^ Thirty different genotypes with 85.7% polymorphism were observed among *Pseudomonas aeruginosa* strains recovered from cystic fibrosis patients in Canada.^28^ In contrast to current study, a low genetic diversity (18%) among 50 isolates of *Pseudomonas aeruginosa* from burn patients was reported from Iran.^29^ This low level of genetic diversity was attributed to cross infection of bacteria among burns patients within the same hospital. Epidemiologically, genetically relatedness of bacterial isolates suggests origin and spread from a common source. The high level of genetic diversity polymorphism in current study could be attributed to location of isolation and diverse clinical specimens used for isolation.

### Limitations of the study

Current study involved sampling for hospitals of Peshawar. A large sampling area could help in better understanding of the epidemiological aspects of resistant strains of *Pseudomonas aeruginosa* at molecular level on regional level.

## CONCLUSION

The high prevalence of metallo beta lactamases genes *blaIMP, blaNDM-1* and *blaVIM* among imipenem resistant strains of *Pseudomonas aeruginosa* is a serious health concern. Strict hygienic conditions, good infection control policies and appropriate use of antibiotics are essential to prevent further dissemination of MBL producing strains of *Pseudomonas aeruginosa*. It is also concluded that genotypic surveillance study must be performed on regular basis to identify possible sources of dissemination and origin of pathogenic bacteria. In this regard, RAPD technique offers low cost and quick method especially in developing countries.

### Author’s Contribution:

**AA:** Did samples collection, conducted experiments and wrote manuscript and responsible and accountable for the accuracy and integrity of the work. **KA:** Designed and supervised the study, analyzed the data and proofread the manuscript. **SR:** Helped in literature search, cooperated during experiments and data interpretation. **IA:** Performed data analysis and proof read the manuscript.
